# Cavernous Malformations of the Central Nervous System: A Comprehensive Review of Pathophysiology, Diagnosis, and Management

**DOI:** 10.7759/cureus.67591

**Published:** 2024-08-23

**Authors:** Kaustuv Das, Jayshree Sen, Aishwarya S Borode

**Affiliations:** 1 Anesthesiology, Jawaharlal Nehru Medical College, Datta Meghe Institute of Higher Education and Research, Wardha, IND; 2 Anaesthesiology, Jawaharlal Nehru Medical College, Datta Meghe Institute of Higher Education and Research, Wardha, IND

**Keywords:** genetic mutations, surgical resection, hemorrhage, neuroimaging, central nervous system (cns), cavernous malformations (cms)

## Abstract

Cavernous malformations (CMs) of the central nervous system (CNS) are vascular anomalies characterized by clusters of dilated, thin-walled blood vessels prone to leakage and hemorrhage. These malformations can occur throughout the CNS, including the brain and spinal cord, and present with a wide range of clinical manifestations, from asymptomatic cases to severe neurological deficits. Advances in neuroimaging, particularly magnetic resonance imaging (MRI), have greatly improved the diagnosis and understanding of CMs, enabling more precise differentiation from other vascular lesions. The management of CMs has evolved alongside advancements in surgical and radiosurgical techniques, offering various therapeutic options depending on the lesion’s characteristics and patient symptoms. While conservative management is often appropriate for asymptomatic or minimally symptomatic lesions, surgical resection or stereotactic radiosurgery may be indicated in cases with recurrent hemorrhage or significant neurological impairment. This comprehensive review explores the pathophysiology, clinical presentation, diagnosis, and management of CMs, highlighting current evidence-based practices and emerging therapeutic approaches. The review also addresses the genetic and molecular underpinnings of CMs, particularly in hereditary cases, and discusses potential future directions in research and treatment. By synthesizing the latest knowledge in the field, this review aims to enhance clinical decision-making and promote further investigation into the optimal management of CMs in the CNS.

## Introduction and background

Cavernous malformations (CMs), or cavernous hemangiomas or cavernomas, are vascular anomalies composed of clusters of abnormally dilated, thin-walled blood vessels [1]. These vessels form irregular, “cavernous” spaces filled with slow-moving or stagnant blood and lined by a single layer of endothelium without any intervening brain tissue. Unlike arteriovenous malformations (AVMs), CMs are not associated with high blood flow and lack significant arterial feeding vessels. The fragile walls of these vessels make them prone to leakage, leading to microhemorrhages or, in some cases, more significant bleeding events [2]. CMs can be found throughout the central nervous system (CNS), including the brain and spinal cord. They are most commonly located in the cerebral hemispheres but can also occur in the brainstem, cerebellum, and spinal cord. These malformations may be solitary or multiple, with multiple lesions often seen in familial cases [3]. The prevalence of CMs in the general population is estimated to be between 0.4% and 0.8%, with cases occurring sporadically or as part of a hereditary syndrome. The hereditary form, which follows an autosomal dominant inheritance pattern, accounts for about 20% of cases and is linked to mutations in one of three genes: *CCM1*, *CCM2*, or *CCM3*. CMs can manifest at any age, though they are typically diagnosed in adults between the third and fifth decades of life [4]. While there is no strong gender preference, some studies suggest a slight predominance in females. Clinically, CMs can range from being completely asymptomatic to causing seizures, focal neurological deficits, or hemorrhagic episodes [5].

The understanding and managing of CMs have undergone significant changes over the past century. Initially, these lesions were frequently misdiagnosed or confused with other types of intracranial hemorrhages or tumors due to the lack of specific diagnostic tools. The term “cavernoma” was introduced in the late 19th century to describe these vascular anomalies, with early knowledge derived primarily from autopsy reports [6]. The introduction of advanced neuroimaging techniques, particularly magnetic resonance imaging (MRI), in the 1980s marked a turning point in diagnosing CMs. MRI allowed for the accurate identification and differentiation of CMs from other vascular lesions, significantly enhancing the understanding of their natural history, clinical manifestations, and hemorrhage risks [7]. Concurrently, treatment strategies evolved as well. Surgical resection was traditionally the primary option, usually reserved for cases with recurrent hemorrhage or intractable seizures. However, with the advent of microsurgical techniques and the development of stereotactic radiosurgery (SRS), the therapeutic landscape for CMs has broadened. These advancements have provided clinicians with a range of treatment options tailored to the lesion’s location, size, and associated symptoms [8].

CMs pose a unique challenge in both neurology and neurosurgery due to their unpredictable nature and potential to cause significant neurological impairment. This comprehensive review is vital as it consolidates current knowledge on CMs, encompassing their pathophysiology, diagnostic approaches, and management strategies. Given the potential of CMs to result in serious neurological consequences, an in-depth understanding of their clinical presentation and management is crucial for optimizing patient outcomes. This review serves as a valuable resource for clinicians, highlighting the most recent evidence-based practices in diagnosing and managing CMs. It guides when to intervene and which treatment modalities may be most appropriate based on individual patient factors. For researchers, the review identifies gaps in the current understanding of CMs. It suggests directions for future research, particularly in unraveling the molecular mechanisms behind these lesions and developing targeted therapies. By integrating a wide array of information, this review aims to enhance the care of patients with CMs and to foster ongoing research in this continually evolving field.

## Review

Pathophysiology

CMs of the CNS are vascular lesions characterized by abnormal clusters of blood vessels. Understanding their pathophysiology involves examining their anatomy, genetic underpinnings, and natural history [9]. Cerebral cavernous malformations (CCMs) consist of dilated, thin-walled vascular channels filled with slow-moving blood. These lesions typically present as irregular, mulberry-like structures and can range in size from a few millimeters to several centimeters. A distinguishing feature of CMs is their lack of intervening neural tissue, setting them apart from other vascular anomalies such as AVMs and developmental venous anomalies (DVAs) [4]. While cerebral and spinal CMs share similar structural characteristics, their clinical presentations and complications can differ. Cerebral CMs are often associated with neurological symptoms, including headaches, seizures, and episodes of hemorrhage. In contrast, spinal CMs may lead to symptoms related to spinal cord compression, such as pain and motor deficits. Due to the risk of neurological impairment, symptomatic spinal lesions are more frequently managed with surgical intervention compared to cerebral CMs, particularly when the latter are asymptomatic [10].

CMs can be classified as either familial or sporadic. Familial, inherited cases often present with multiple lesions, whereas sporadic cases typically manifest as single malformations without a family history. Approximately 85% of cases are sporadic, while about 15% are familial, indicating a significant genetic component in the latter [11]. Three primary genes are implicated in familial CMs: CCM1 (KRIT1), CCM2, and CCM3 (PDCD10). Mutations in CCM1 are the most common cause of familial CCMs and are known to affect vascular integrity. Similarly, mutations in CCM2 and CCM3 also contribute to the pathogenesis of familial CMs by disrupting the normal functioning of endothelial cells and the structural integrity of blood vessels [12]. The molecular pathways involved in CM development include those regulating cell adhesion, vascular development, and endothelial cell function. Genetic mutations that disrupt these pathways lead to the formation of abnormal vascular structures, resulting in the clinical manifestations associated with CMs [13].

The risk of hemorrhage in patients with CMs is a significant concern, particularly for those with a history of previous bleeding. Hemorrhages can result in severe neurological deficits and complications, such as hemorrhagic stroke. The risk of recurrent bleeding is notably higher in CMs located in the brainstem and those that have already experienced a hemorrhage [14]. The natural history of CMs varies; some CMs may remain stable or regress over time, while others can grow or lead to complications. Long-term outcomes depend on factors including the malformation’s location, symptoms’ presence, and management approach [15]. Regular imaging is often recommended for asymptomatic patients, while symptomatic cases may require intervention. In summary, CMs of the CNS present a complex interplay of anatomical features, genetic predispositions, and variable clinical outcomes, necessitating a comprehensive understanding of effective management and treatment strategies [15].

Clinical presentation

CMs can present with a range of symptoms depending on their location within the CNS and whether they are asymptomatic or symptomatic. CCMs may manifest with various neurological symptoms, including recurrent headaches, seizures, and focal neurological deficits [16]. Headaches are often recurrent and can vary in intensity, potentially caused by irritation of surrounding tissues or hemorrhage. Seizures are common, especially in patients with lesions in cortical regions, and may be focal or generalized. Additionally, depending on the location of the CM, patients might experience neurological deficits such as weakness, sensory loss, or speech difficulties due to direct compression of adjacent brain structures or hemorrhagic events [17]. In contrast, spinal CMs present with symptoms related to their location within the spinal column. Patients often report localized back pain or radicular pain radiating along nerve roots. Depending on the level of the spinal cord affected, motor and sensory disturbances, such as weakness, numbness, or tingling in the extremities, may occur. Severe cases can lead to significant functional impairment, underscoring the need for timely diagnosis and management [18].

Many CMs are found incidentally during imaging for unrelated issues, raising questions about their clinical significance. Asymptomatic CMs may not require immediate intervention, but their potential for future hemorrhage necessitates careful monitoring. Although some asymptomatic lesions can remain stable for years, there is always a risk of sudden symptom onset due to hemorrhage. Regular follow-up imaging may be recommended to monitor changes and allow timely intervention if needed [19]. Symptomatic CMs typically present with a sudden change in neurological status, often due to hemorrhage or progressive neurological deficits. Management of symptomatic CMs is more urgent and may involve surgical intervention to alleviate symptoms and prevent further complications. Understanding the risk factors influencing symptom development in CMs is crucial for effective risk stratification and management [20].

**Figure 1:**
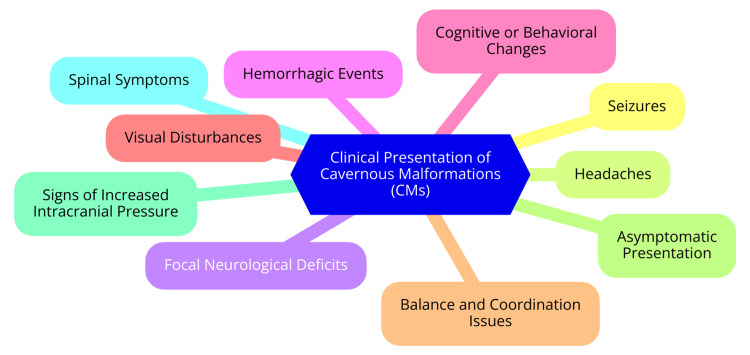
Clinical presentation of CMs CMs: cavernous malformations Image credit: Dr Aishwarya S. Borode

Several factors can increase the likelihood of hemorrhage in patients with CMs, including the size of the malformation, its location, and a history of previous hemorrhage. Larger CMs generally have a higher risk of bleeding compared to smaller lesions, and those located in eloquent areas of the brain or near critical vascular structures pose an even greater risk for symptomatic hemorrhage. Furthermore, a history of prior bleeding events significantly elevates the risk of subsequent hemorrhagic episodes, highlighting the importance of careful monitoring and management [16]. The specific anatomical location of the CM influences the type and severity of symptoms experienced. For example, brainstem or spinal cord lesions may lead to more severe neurological deficits than cerebral cortex ones. Additionally, the patient’s genetic background can affect symptom development. Familial forms of CMs, particularly those associated with genetic mutations (e.g., KRIT1, CCM2, PDCD10), may present differently and carry varying risks for symptom development. In such cases, genetic counseling may provide valuable information and support for affected families regarding the implications of these genetic factors [21]. The clinical presentation of CMs is illustrated in Figure 1.

Diagnosis

Imaging Techniques

MRI is the gold standard for diagnosing CMs due to its superior soft tissue contrast and ability to provide detailed images of the CNS. Key MRI sequences, including T1-weighted and T2-weighted images, are essential for identifying CMs [22]. On T1-weighted images, CMs typically appear as hypointense (dark) lesions, while T2-weighted images show them as hyperintense (bright) due to blood products and hemosiderin. Gradient echo (GRE) sequences are particularly useful for detecting hemosiderin deposits around the CM, which indicate prior hemorrhages. Although MRI is the preferred imaging modality, other techniques can also be useful [23]. Computed tomography (CT) scans can detect acute hemorrhages associated with CMs, revealing hyperdense areas due to the presence of blood; however, they are less sensitive than MRI for identifying the malformations themselves. Digital subtraction angiography (DSA) is primarily employed to evaluate vascular lesions but has limited utility in diagnosing CMs, as these lesions generally do not exhibit significant vascular supply. In certain cases, particularly pediatric patients, ultrasound may be used, although it is not a standard diagnostic tool for CMs [24].

Imaging Characteristics

CMs exhibit distinct characteristics across different imaging modalities. On MRI, CMs typically appear as well-defined, round, or lobulated lesions with a “popcorn” appearance due to multiple small blood-filled spaces. A surrounding hemosiderin rim, indicative of previous hemorrhages, is a key feature of CMs [25]. On CT scans, CMs may present as low-density lesions, particularly in chronic cases, while acute hemorrhage can show as hyperdense areas. However, the malformation itself might not be visualized. Angiography is less effective for diagnosing CMs because these lesions generally do not have a significant vascular supply, unlike other vascular anomalies such as AVMs. AVMs are characterized by a “nidus” appearance on angiography and exhibit abnormal arterial and venous connections, which are absent in CMs. DVAs may coexist with CMs but can be distinguished by their “caput medusae” appearance on imaging. Hemangiomas might also resemble CMs but typically appear more vascular [26].

Diagnostic Challenges

Despite advancements in imaging technology, several limitations persist in diagnosing CMs. Although MRI is highly sensitive, it may still miss small or deep-seated CMs, and the presence of other lesions can complicate the interpretation. Motion artifacts or susceptibility artifacts from adjacent structures can obscure the visualization of CMs on MRI. Additionally, variability in imaging protocols between institutions can result in differences in the detection and characterization of CMs [22]. A thorough differential diagnosis is crucial to avoid misinterpretation of imaging findings. Conditions to consider include neoplasms such as gliomas or metastases, which can mimic the appearance of CMs, and ischemic changes that may present similarly, especially in the acute phase. Other vascular lesions, including AVMs and DVAs, must also be considered [27]. Table 1 illustrates the diagnostic challenges associated with CMs.

**Table 1:** Diagnostic challenges in cavernous malformations CMs: cavernous malformations; MRI: magnetic resonance imaging

Diagnostic Challenge	Description	Impact on Diagnosis	Potential Solutions	Current Status
Non-specific symptoms	Symptoms of CMs can be similar to other neurological disorders, such as seizures or headaches.	This may lead to misdiagnosis or delayed diagnosis.	Comprehensive clinical evaluation; differential diagnosis.	Ongoing refinement of diagnostic criteria and clinical guidelines.
Variable imaging appearance	CMs can vary in appearance on different imaging modalities, complicating identification.	Difficulty in distinguishing CMs from other vascular lesions.	Use of advanced imaging techniques like high-resolution MRI.	Continuous development in imaging technology.
Small lesion size	Small CMs may be difficult to detect, especially in lower-resolution imaging studies.	Risk of missing small lesions that could lead to significant symptoms.	Enhanced imaging techniques; repeat imaging if suspicion remains.	Ongoing research into more sensitive imaging modalities.
Hemorrhage and acute changes	Active hemorrhage or changes in CM size can alter imaging findings, making diagnosis challenging.	It may obscure the CM or mimic other conditions.	Timely imaging follow-up; consideration of clinical history.	Improved imaging protocols and follow-up strategies.
Overlapping features with other vascular malformations	CMs may be confused with other conditions, such as arteriovenous malformations (AVMs) or venous angiomas.	Challenges in differentiating between types of vascular malformations.	Detailed imaging analysis; use of contrast agents.	Enhanced diagnostic criteria and imaging techniques.
Genetic testing limitations	Limited availability or accessibility of genetic testing for hereditary forms of CMs.	Delays in identifying genetic causes, especially in familial cases.	Expanding access to genetic testing; increased awareness.	Increasing availability of genetic testing options.
Patient variability	Variability in how CMs present among different patients, including asymptomatic cases.	Difficulty in establishing a standardized diagnostic approach.	Personalized diagnostic and monitoring strategies.	Growing recognition of the need for individualized approaches.
Radiologist expertise	Variability in the interpretation of CM imaging among radiologists with varying levels of expertise.	Potential for diagnostic inconsistency or error.	Specialized training and experience for radiologists.	Development of training programs and diagnostic standards.

Management

Observation and Conservative Management

Observation is generally recommended for asymptomatic CMs or those with minimal symptoms. Indications for monitoring include incidental findings on imaging without neurological deficits, stable patients with a history of seizures or hemorrhage, and familial cases with multiple CMs that are not symptomatic [28]. For patients under observation, follow-up typically involves annual MRI scans to evaluate any changes in lesion size, number, or the presence of new microhemorrhages. Regular neurological evaluations are crucial to detect new symptoms or deterioration in existing conditions [29].

Surgical Management

Surgical intervention is indicated for CMs that are symptomatic or pose a risk of significant complications. Surgery is considered for severe or recurrent hemorrhages, intractable seizures associated with the CM, and progressive neurological deficits that significantly affect quality of life. The choice of surgical technique depends on the location and size of the malformation [8]. Microsurgical resection is the standard approach for accessible lesions, especially in non-eloquent brain areas. Endoscopic techniques may sometimes be used depending on the lesion’s location. Surgical outcomes are generally favorable, with low recurrence rates for hemorrhage (approximately 0.4%-7.7%) and minimal neurological deficits. Nonetheless, potential complications include infection, neurological deficits resulting from the surgical approach, and rebleeding, particularly if the resection is incomplete [30].

Radiosurgery

SRS serves as an alternative to microsurgery, especially for lesions that are difficult to access or in patients who are not candidates for traditional surgery. Indications for SRS include lesions located in eloquent brain areas where surgical risks are elevated and patients with multiple CMs for whom surgical intervention may not be feasible. The efficacy of SRS is still being assessed, with optimal results often observed after a latency period of up to 2 years. Risks associated with SRS include radiation-induced changes, potential for delayed hemorrhage, and neurological deficits. However, these risks are generally lower than traditional surgery [31]. Table 2 outlines the management strategies for the CNS’s CMs.

**Table 2:** Management of cavernous malformations in the central nervous system CMs: cavernous malformations; MRI: magnetic resonance imaging

Management Approach	Indications	Advantages	Limitations/Considerations
Conservative management	Asymptomatic CMs	Avoids surgical risks	Requires regular monitoring with MRI
	Minimally symptomatic CMs	Suitable for low-risk lesions	Risk of future hemorrhage
Surgical resection	Symptomatic CMs with recurrent hemorrhage	Definitive removal of the lesion	Surgical risks, including neurological deficits
	Lesions causing significant neurological deficits	Reduces the risk of future hemorrhage	Accessibility depends on the lesion location
	Superficial and accessible lesions	Potential for symptom resolution (e.g., seizure control)	Requires experienced surgical expertise
Stereotactic radiosurgery	Deep-seated or inoperable lesions	Minimally invasive	Risk of radiation-induced complications
	Lesions with high surgical risk	Targeted treatment with reduced recovery time	Delayed response to treatment
	Recurrent hemorrhages in surgically inaccessible areas	It can be used in conjunction with conservative management	Limited long-term data on efficacy
Pharmacological management	Seizure control in patients with CMs	Reduces seizure frequency	Does not address the underlying lesion
	Symptomatic management (e.g., anti-epileptic drugs)	Non-invasive, easily administered	Long-term medication use may be required
Follow-up and monitoring	All patients with diagnosed CMs	Regular monitoring allows for timely intervention	Requires adherence to follow-up schedule
	Post-surgical or radiosurgical patients	Can detect lesion changes or new hemorrhages	MRI availability and cost

Emerging Therapies and Future Directions

Research actively explores pharmacological therapies to reduce the risk of bleeding from CMs. These investigational treatments include anti-angiogenic agents and other medications designed to target the vascular structures of the malformations. Advances in genetic research also hold promise for targeted therapies that address the molecular pathways involved in CM development. Such approaches may lead to more effective management strategies tailored to individual patient profiles, especially in familial cases with a known genetic predisposition [32]. Table 3 presents emerging therapies and future directions for treating CMs.

**Table 3:** Emerging therapies and future directions in the management of cavernous malformations CMs: cavernous malformations

Therapy/Direction	Description	Current Status	Potential Benefits	Challenges/Considerations
Targeted molecular therapy	Exploring drugs targeting specific molecular pathways involved in CM formation and progression.	Early-stage research and clinical trials.	It may prevent lesion growth or reduce hemorrhage risk.	Identifying effective targets, safety, and efficacy concerns.
Gene therapy	Techniques aimed at correcting genetic mutations in hereditary forms of CMs (e.g., CCM1, CCM2, and CCM3).	Experimental; preclinical studies ongoing.	Potential to cure or prevent familial CMs.	Delivery methods; long-term effects; ethical considerations.
Immunotherapy	Investigating immune modulation to reduce inflammation associated with CM hemorrhage.	Experimental phase; animal models.	It may reduce bleeding events and improve lesion stability.	Immune-related side effects need more human trials.
Advanced imaging techniques	Development of high-resolution imaging modalities for better CM characterization and risk assessment.	Clinical studies in progress.	Improved diagnosis, monitoring, and surgical planning.	High cost; accessibility in routine clinical practice.
Minimally invasive surgical techniques	Enhancements in endoscopic and robotic-assisted surgeries to remove CMs with minimal tissue damage.	Ongoing refinement and adoption.	Reduced postoperative morbidity; faster recovery.	Technical complexity requires specialized training.
Stereotactic radiosurgery advances	Refinement of radiosurgery techniques, including dose modulation and precision targeting.	Clinical application with continuous improvements.	Non-invasive option for difficult-to-reach CMs.	Risk of radiation-induced complications; long-term outcomes.
Biomarker discovery	Identifying blood or cerebrospinal fluid biomarkers for early detection and monitoring of CMs.	Research and validation phases.	Non-invasive monitoring; personalized treatment strategies.	Validation in larger populations; sensitivity and specificity issues.
Artificial intelligence (AI) in diagnosis	Implementing AI algorithms to enhance CM detection and risk stratification in imaging.	Early adoption in some centers; research ongoing.	Enhanced diagnostic accuracy; predictive analytics.	Data privacy concerns: need for large datasets for training.
Combination therapies	Exploring the synergistic effects of combining molecular, immuno-, and radiosurgery therapies.	Research and clinical trials in early stages.	Potential for more effective and comprehensive treatment.	Complexity of regimen; managing combined side effects.
Patient-specific treatment planning	Use 3D modeling and personalized medicine approaches to tailor treatment plans for individual patients.	Emerging practice with increasing interest.	Optimized outcomes; reduced unnecessary interventions.	Resource-intensive; requires multidisciplinary collaboration.

Prognosis and long-term outcomes

Predictors of Outcomes

Several factors significantly influence the long-term prognosis of patients with CMs. One of the most critical factors is the location and size of the malformation. CMs located in eloquent areas of the brain - regions responsible for essential functions such as language and motor control - tend to have poorer outcomes than those in non-eloquent regions. Additionally, larger malformations are associated with an increased risk of complications and less favorable functional recovery following surgical intervention [33]. Another important predictor is the patient’s preoperative neurological status, often assessed using the modified Rankin Scale (mRS), which measures the degree of disability or dependence on daily activities. Patients with a higher preoperative mRS score, indicating greater disability, are more likely to experience less favorable outcomes after treatment. Furthermore, a history of hemorrhage is a crucial factor in prognosis; patients with multiple bleeding episodes may face a higher risk of neurological deterioration and complications [34]. The impact of treatment on outcomes is also significant. Surgical resection remains the primary treatment for symptomatic CMs, especially those that have bled. Research indicates that surgical intervention often substantially improves symptoms and functional status. However, the risk of complications, such as rebleeding and neurological deficits, persists, particularly for lesions located in challenging areas like the brainstem. Despite these risks, long-term outcomes following surgery are generally positive, with many patients reporting enhancements in their quality of life and neurological function. Nonetheless, some individuals may experience persistent symptoms, requiring ongoing management and rehabilitation [35].

Quality-of-Life Considerations

CMs can significantly impact a patient’s quality of life. Symptoms such as seizures, headaches, and neurological deficits often restrict daily activities and can lead to emotional distress. Many patients experience anxiety and depression related to their condition, underscoring the need to address both physical and psychological aspects of care. Research utilizing quality-of-life assessment tools, such as the Short Form-12 questionnaire, indicates that while many patients report improved quality of life after surgery, those with CMs in eloquent brain areas may still experience reduced health perceptions compared to normative data. This suggests that even with surgical success, the location and effects of the malformation can result in enduring quality-of-life challenges [36]. Rehabilitation is a vital component of the recovery process for patients with CMs. Physical therapy can aid in restoring strength and function, while psychological support helps patients cope with the emotional burden of living with a chronic condition. Support groups and counseling offer valuable resources for patients and their families, fostering a sense of community and shared experience. The prognosis and long-term outcomes for patients with CMs are influenced by factors such as lesion characteristics, preoperative status, and treatment approaches. Quality-of-life considerations are crucial, necessitating a comprehensive approach that includes rehabilitation and supportive care to address physical and emotional needs [37].

Special considerations

Pediatric Cavernous Malformations

Pediatric CMs present distinct challenges in terms of presentation, diagnosis, and management compared to those in adults. In children, CMs often manifest with different clinical symptoms, including seizures, headaches, and focal neurological deficits. Research has shown that a significant proportion of pediatric patients with CMs experience seizures, while others report headaches. In some cases, multiple CMs can further complicate management [38]. MRI remains the primary imaging modality for diagnosing CMs in children. However, the imaging characteristics of pediatric CMs may differ from those observed in adults, requiring careful interpretation by specialists experienced in pediatric cases. Genetic counseling and testing are also essential, particularly for families with a history of CMs, as understanding the genetic basis can significantly impact management decisions [39]. In terms of treatment, surgical resection is often the preferred approach for symptomatic solitary CMs in children. Evidence indicates that a high percentage of children undergoing surgery achieve complete resection and substantial improvement in seizure control. For asymptomatic or multiple CMs, a conservative management strategy involving regular monitoring is generally recommended, allowing for timely intervention if symptoms develop [40].

CMs in Pregnancy

Managing CMs during pregnancy requires meticulous attention to both maternal and fetal health. Pregnant women with known CMs should be closely monitored, especially if they have a history of hemorrhage or neurological symptoms. The physiological changes during pregnancy, such as increased blood volume and hormonal fluctuations, may heighten the risk of hemorrhage, making careful observation crucial [41]. Regarding management strategies, conservative approaches are generally preferred unless the CM becomes symptomatic. Surgical intervention during pregnancy is typically avoided unless necessary due to the potential risks to both the mother and fetus. The possibility of hemorrhage and neurological deficits presents challenges that must be carefully balanced against the benefits of any surgical procedure [42].

CMs Associated with Other Conditions

CMs often coexist with other vascular malformations or neurological disorders, which can complicate their management. For example, CMs are frequently found alongside DVAs, complicating both diagnosis and treatment strategies. Multiple vascular lesions necessitate a multidisciplinary approach involving neurosurgeons, neurologists, and radiologists to provide comprehensive care [43]. Additionally, patients with CMs may have other neurological disorders, such as epilepsy or developmental delays. This comorbidity requires a thorough evaluation to create treatment plans that address all aspects of the patient’s health. Coordinated care is essential to manage the complexities associated with CMs and their potential interactions with other medical conditions, ensuring the best possible outcomes for patients [44].

## Conclusions

In conclusion, CMs of the CNS represent a complex and often challenging condition characterized by their unpredictable clinical course and potential for significant neurological morbidity. Advances in neuroimaging, particularly with the widespread use of MRI, have greatly improved our ability to diagnose and understand the natural history of these vascular anomalies. Despite these advancements, the management of CMs remains nuanced, requiring a careful balance between the risks of intervention and the potential for spontaneous hemorrhage or neurological decline. Surgical resection, radiosurgery, and conservative monitoring each have their place in the therapeutic arsenal, with decisions tailored to individual patient circumstances. As our understanding of the genetic and molecular underpinnings of CMs deepens, there is hope for developing more targeted and effective therapies. This comprehensive review underscores the importance of continued research and clinical vigilance in optimizing outcomes for patients with CMs while also highlighting the need for personalized care strategies that consider the unique characteristics of each lesion and patient.
